# Externally‐Led Patient‐Focused Drug Development Meeting (EL‐PFDD) for Dementia with Lewy Bodies—DLB: Voices of Lived Experience on Life with Lewy and Unmet Treatment Needs

**DOI:** 10.1002/alz70859_104394

**Published:** 2025-12-25

**Authors:** Keith N. Fargo, Melissa J. Armstrong, Larry J. Bauer, Brad F Boeve, James E. Galvin, Marwan N. Sabbagh, James E. Valentine, Sam Fazio, Guttmacher Laurence, Barbara Levine, Helen Bundy Medsger, Lycia Tramujas Vasconcellos Neumann, Kimberly Pfleeger, Glorieuse Pugh, Elizabeth Rachchh, Jonathan Stokes, Deborah Walter, Angela S. Taylor

**Affiliations:** ^1^ Lewy Body Dementia Association, Lilburn, GA USA; ^2^ University of Florida, Gainesville, FL USA; ^3^ Hyman, Phelps & McNamara, P.C., Washington, DC USA; ^4^ Mayo Clinic, Rochester, MN USA; ^5^ University of Miami Miller School of Medicine, Boca Raton, FL USA; ^6^ Barrow Neurological Institute, Phoenix, AZ USA; ^7^ Alzheimer's Association, Chicago, IL USA; ^8^ Living with DLB, and University of Rochester School of Medicine, Rochester, NY USA; ^9^ Care partner for person living with DLB, Buffalo Grove, IL USA; ^10^ Care partner for person living with DLB, Santa Rosa, CA USA; ^11^ AbbVie, North Chicago, IL USA; ^12^ GE HealthCare, Chicago, IL USA

## Abstract

**Background:**

Dementia with Lewy bodies (DLB) is a prevalent form of dementia associated with neuronal alpha‐synuclein aggregates (Lewy bodies). Symptoms include cognitive impairment, fluctuations, neuropsychiatric features including psychosis, sleep disorders, and dysautonomia. DLB is the most expensive form of dementia, and there are currently no treatments approved by the US FDA. Given the advent of sensitive and specific in vivo biomarkers for synuclein aggregation, drug developers are increasingly interested in DLB. FDA instituted Patient‐Focused Drug Development (PFDD) meetings in 2012 with the purpose of ensuring that patients’ experiences, perspectives, needs, and preferences are captured and meaningfully incorporated into drug development and evaluation.

**Method:**

In October 2024, the Lewy Body Dementia Association (LBDA) and the Alzheimer’s Association (ALZ) co‐hosted an externally‐led PFDD (EL‐PFDD) meeting on DLB. Thirty days before the event, LBDA solicited public comment on the lived experience of DLB, the current state of treatment, and what people with DLB value in potential future therapies. During the live meeting, people with DLB, care partners and family members described their experiences and responded to live polling questions.

**Result:**

The EL‐PFDD was attended by 347 people with DLB and care partners, drug developers (both industry and academic), FDA staff, and NIH staff. Preliminary analysis of the discussion content and survey responses shows several themes emerging from the meeting. The most bothersome and impactful symptoms varied over the course of the disease, but included cognitive impairment and fluctuations, neuropsychiatric symptoms, and sleepiness. Concerns for the future included progressive decline in cognition, changes in behavior/mood, and cost of care. Limitations to current treatments included lack of FDA‐approved medications, and side effects and lack of efficacy associated with “off label” medications. Priorities for future treatments included symptomatic treatments that do not worsen other DLB symptoms, slowing disease progression, accessibility/insurance coverage, and avoiding side effects.

**Conclusion:**

The EL‐PFDD on DLB provided valuable information for drug developers and regulators to understand the lived experience of people with DLB and care partners. The results will be documented in a “Voice of the Patient” report that will be hosted at www.lbda.org/el‐pfdd.

